# Inflammatory Mediators in Obesity

**DOI:** 10.1155/2019/9481819

**Published:** 2019-07-31

**Authors:** Divya P. Kumar, Saisudha Koka, Chao Li, Senthilkumar Rajagopal

**Affiliations:** ^1^Department of Biochemistry, JSS Medical College, CEMR, JSS Academy of Higher Education and Research, Mysore, Karnataka, India; ^2^College of Pharmacy, University of Houston, Houston, Texas, USA; ^3^Department of Internal Medicine, Virginia Commonwealth University, Richmond, Virginia, USA; ^4^Department of Biochemistry, Rayalaseema University, Kurnool, Andra Pradesh, India

The epidemic of obesity is increasing exponentially, and nearly a third of the world's population is now either overweight or obese [1]. The prevalence of obesity is higher in women than men and increases with age. There is also an alarming increase in the incidence of obesity in children and adolescent [2]. Although there is genetic influence on body weight, obesity results from complex interactions of many factors, including life style, metabolic, socioeconomic, and environmental factors. Thus, obesity, the accumulation of excessive fat, adversely affects nearly all the physiological functions of the body and has emerged as a serious public health challenge [3].

Obesity-induced inflammation is marked by an increased number and activation of immune cells, including macrophages, neutrophils, and T helper cells, leading to the production of proinflammatory cytokines such as tumor necrosis factor *α* (TNF-*α*), interleukins, and C-reactive proteins (CRP) while simultaneously suppressing anti-inflammatory cells and reducing production of adiponectin, predisposing to various cellular stresses like endoplasmic reticulum (ER) stress, mitochondrial dysfunction, and oxidative stress [4]. Obesity-induced inflammation has been implicated as a risk factor in the pathogenesis of insulin resistance, type 2 diabetes mellitus (T2DM), cardiovascular diseases, and metabolic syndrome. It is also associated with the development of other diseases such as psoriasis, renal diseases, polycystic ovary syndrome (PCOS), and cancer [5].

This special issue features a clinical study, original research articles, and review articles that provide insights on various inflammatory markers in obesity-associated diseases. This topic had 21 manuscripts submitted, among which only 9 were accepted for publication.

R. Gomez-Huelgas and colleagues have investigated the impact of intensive life style modification on the levels of adipokine and inflammatory biomarkers in metabolically healthy obese women. The study was a 2-year personalized intervention related to life style modification including a calorie-restricted Mediterranean diet and physical exercise. S. J. Sidles et al. determined how high-fat diet (HFD) alters immunogenic properties of circulating and myeloid-derived CD45^+^ DDR2^+^ cells in adipose tissue. They have analyzed myeloid-derived CD45^+^ DDR2^+^ cells and CD4^+^ T cells from peripheral blood (PB), mammary gland-associated adipose tissue (MGAT), and visceral adipose tissue (VAT). They found that myeloid-derived CD45^+^ DDR2^+^ cells were more activated in the adipose tissue of HFD-fed preobese mice promoting Th1-type skewing and the production of inflammatory cytokines. The immune system is known to play a key role in the development and progression of T2DM and is characterized by the alterations in the profile of circulating immune cells. M. Šiklová and colleagues have examined the circulating monocyte and lymphocyte populations in association with genetic predisposition to T2DM and the response of these cells to short-term hyperinsulinemia in healthy first-degree relatives of T2D when compared to control subjects. The authors, in addition to providing evidence that there exists an interplay between immune system homeostasis and insulin levels, also showed that there are alterations of the CD4/CD8 lymphocyte ratio, relative content of Th17 cells, and intermediate monocytes in FDR signifying the role of the immune system in the pathogenesis of T2DM. Furthermore, studies by E. Dozio et al. elucidated the role of the receptor for advanced glycation end products (RAGE) in lipid accumulation in the heart of obese Zucker rats. The authors showed that increased levels of sRAGE (soluble), especially esRAGE (endogenous secretory form), might protect against obesity-induced intromyocardial lipid accumulation by preventing RAGE hyperexpression, therefore allowing lipids to be metabolized. L. Elizondo-Montemayor and colleagues have investigated the concentration of Irisin, a myokine, and its association with high-sensitivity C-reactive proteins (hs-CRP) as well as with metabolic and anthropometric parameters in children and adolescents with T2DM compared to healthy controls. They have explained the possible Irisin-inflammatory crosstalk in overt T2DM and exacerbation of metabolic derangements due to hypoirisinemia emphasizing the need of a detailed study to better understand the mechanisms involved.

In the study by A. R. Kolodziej et al., the authors have performed a systematic review and meta-analysis to explore the prognostic role of elevated myeloperoxidase (MPO) in patients with acute coronary syndrome (ACS). They observed that high MPO levels were associated with the risk of mortality in ACS patients and hence recommended incorporating MPO in risk stratification models that guide therapy of high-risk ACS patients. J. Zhao et al. have elicited the mechanisms of how podocyte injury is caused in obesity-related glomerulopathy (ORG). The authors have showed that in the pathogenesis of ORG, increased expression of CD36 promotes lipid accumulation and activation of NLRP3 inflammasome leading to the secretion of inflammatory cytokines, which causes the injury of podocytes. C. Rodríguez-Cerdeira and colleagues have reviewed the literature about the biomarkers of inflammation in obesity-psoriatic patients. The data available so far strongly suggested that the inflammatory state associated with obesity is a predisposing factor for the development of psoriasis and that obesity aggravates the existing psoriasis. The review summarizes the diagnostic, prognostic, and treatment response biomarkers of inflammation in obesity-psoriatic patients. In the clinical study, D. Pu et al. revealed that the levels of serum ANGPTL8 were elevated in PCOS patients with metabolic syndrome relative to those without metabolic syndrome and this was associated with insulin resistance and adiponectin levels.

Taken together, this special issue aims to emphasize the critical need for the development of effective therapeutic interventions targeting inflammatory pathways in obesity.
